# Supplementary material for: High resolution ensemble description of metamorphic and intrinsically disordered proteins using an efficient hybrid parallel tempering scheme
Source: Nat Commun. 2021 Feb 11;12:958. doi: 10.1038/s41467-021-21105-7 (PMC7878814; doi:10.1038/s41467-021-21105-7)
Supplement: Supplementary file 1 — Supplementary Information [file 41467_2021_21105_MOESM1_ESM.pdf]

Supporting information

High resolution ensemble description of metamorphic and intrinsically disordered proteins using an efficient hybrid parallel tempering scheme

Authors: Rajeswari Appadurai[1], Jayashree Nagesh[2] and Anand Srivastava[1*]

Affiliations:

[1]Molecular Biophysics Unit, Indian Institute of Science, C.V. Raman Road, Bangalore, Karnataka-560 012, India

[2] Solid State & Structural Chemistry Unit, Indian Institute of Science, C.V. Raman Road, Bangalore, Karnataka-560 012, India

Email: anand@iisc.ac.in
ORCID Id: 0000-0002-2757-1511

Supplementary Table 1: System descriptions and details of the replica exchange simulation parameters.

System studied	Simulation Method	Number of atoms	Number of replicas	Overall Effective temperature range	λ scaling and bath temperature	Cumulative time (in ns)	Average exchange acceptance probability	Transit time# (in ns)
Alanine dipeptide	REHT	1864	5	300–600K	λ=1-0.567, T=300-340K	100	0.25	6
	REST2		3		λ=1-0.5, T=300K	60	0.30	4
Trp-cage	REHT	21038	12	300–478K	λ=1-0.71, T=300-340K	12000	0.12	10
	REST2		8		λ=1-0.63, T=300K	8000	0.30	2
Beta-hairpin	REHT	17203	12	300–478K	λ=1-0.71, T=300-340K	12000	0.12	5
	REST2*	10222	10	300–572K	λ=1-0.52, T=300K	-	0.15–0.40	-
Histatin-5	REHT	23195	15	300–478K	λ=1-0.71, T=300-340K	11250	0.15	3
	REST2		10		λ=1-0.63 T=300K	7500	0.45	1
RFA-H	REHT	40861	25	310-705K	λ=1-0.48, T=310-340K	25000	0.17	110
	REST2		25		λ=1-0.44 T=310K	6250	0.45	Not achieved till 250ns

* In case of Beta-hairpin the REHT simulation is compared with the REST2 simulation performed earlier (Ref.[1]) using the same forcefield.

# Transit time indicates the time taken for a replica to jump over the complete state space. The complete replica mixing allows the full utilization of the temperature space and provides sufficient energy for a biomolecule to undergo conformational transition across the barrier. However, in complex systems like RFA-H, the REST2 method is shown to be ineffective where the complete mixing of replicas is not achieved.

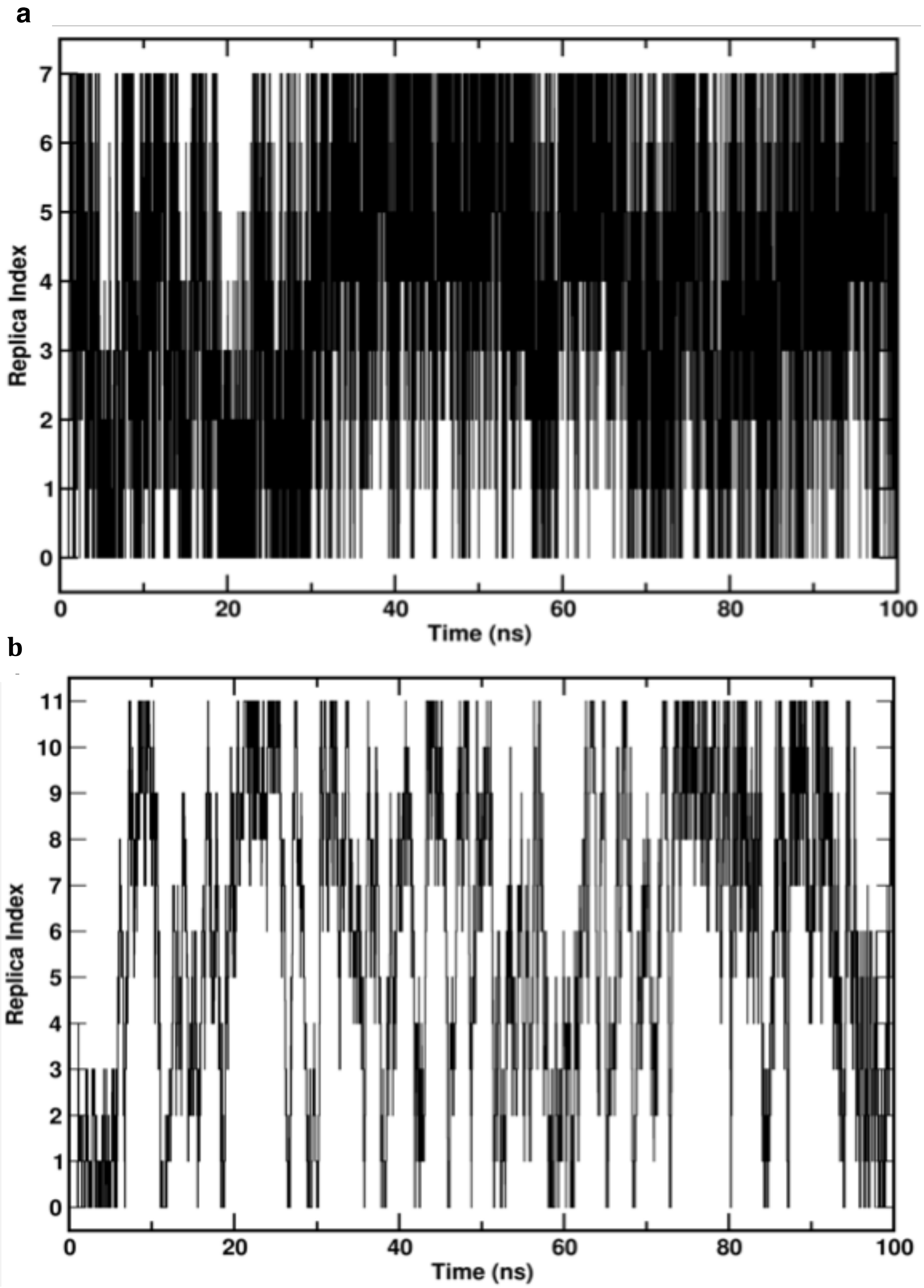

Supplementary Figure 1: Exchange of base replica (rep 0) across the complete replica state space in TRP-cage system simulated with **a**) REST2 and **b**) REHT method. For the sake of clarity only the first 100 ns data are shown.

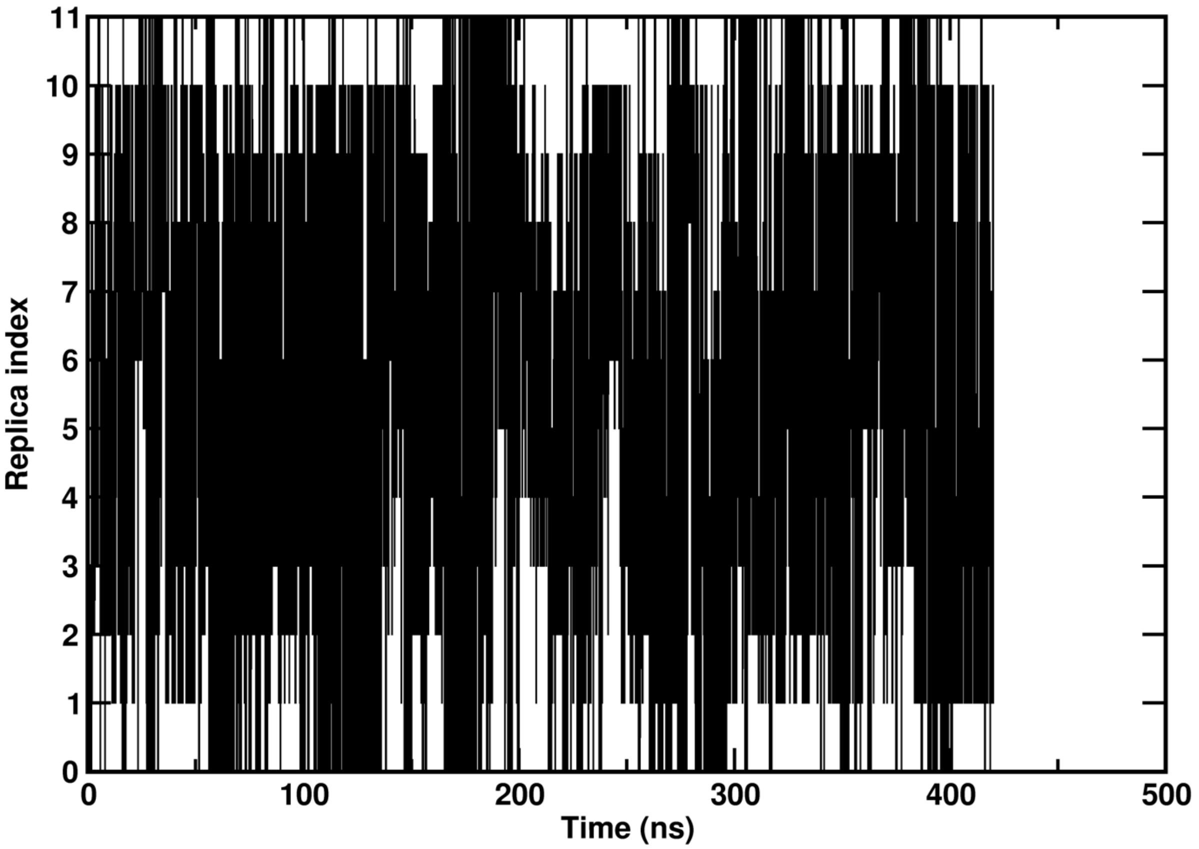

Supplementary Figure 2:Exchange of base replica (rep 0) across the entire replica state space in beta hairpin system simulated with REHT method. The corresponding simulation of the peptide with the REST2 scheme using the same force field can be found in Ref.[1]

a

b

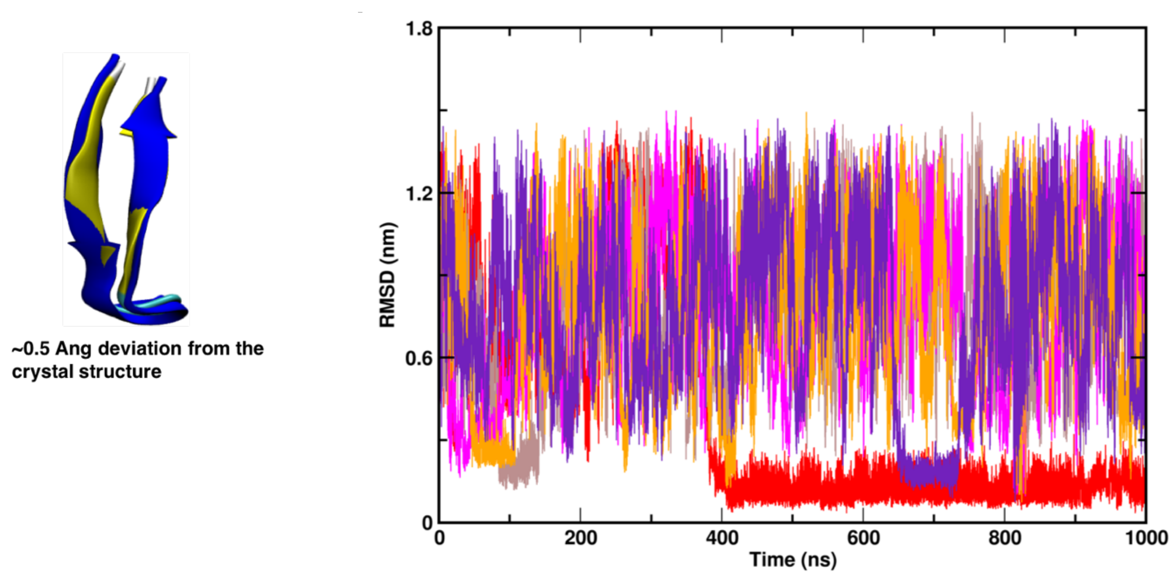

Supplementary Figure 3: **a**) Structural overlay of the simulation-generated beta hairpin (yellow) on the native NMR structure (blue). **b**) Time evolutions of backbone RMSD of REHT-generated Beta hairpin structures with reference to the corresponding NMR structure (1le3), shown for the five (time-continuous) replicas that are successfully folded.

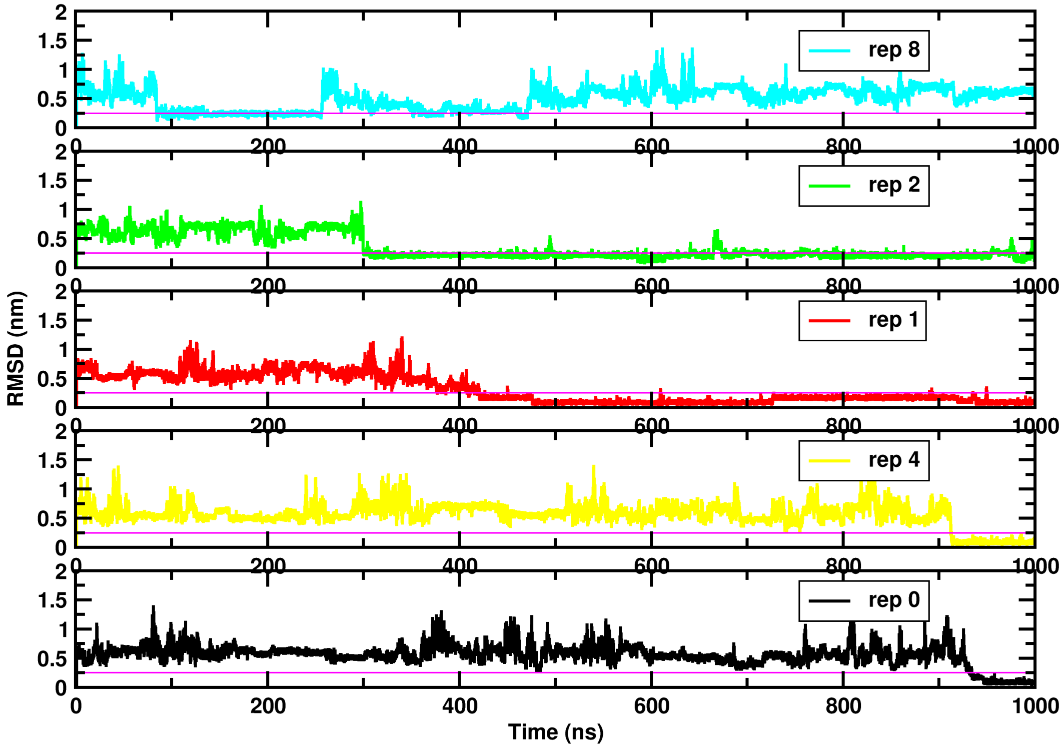

Supplementary Figure 4: Time evolutions of backbone RMSD of REHT-generated TRP-cage structures with reference to the native NMR structure (1l2y). All the five successfully folded replicas are shown.

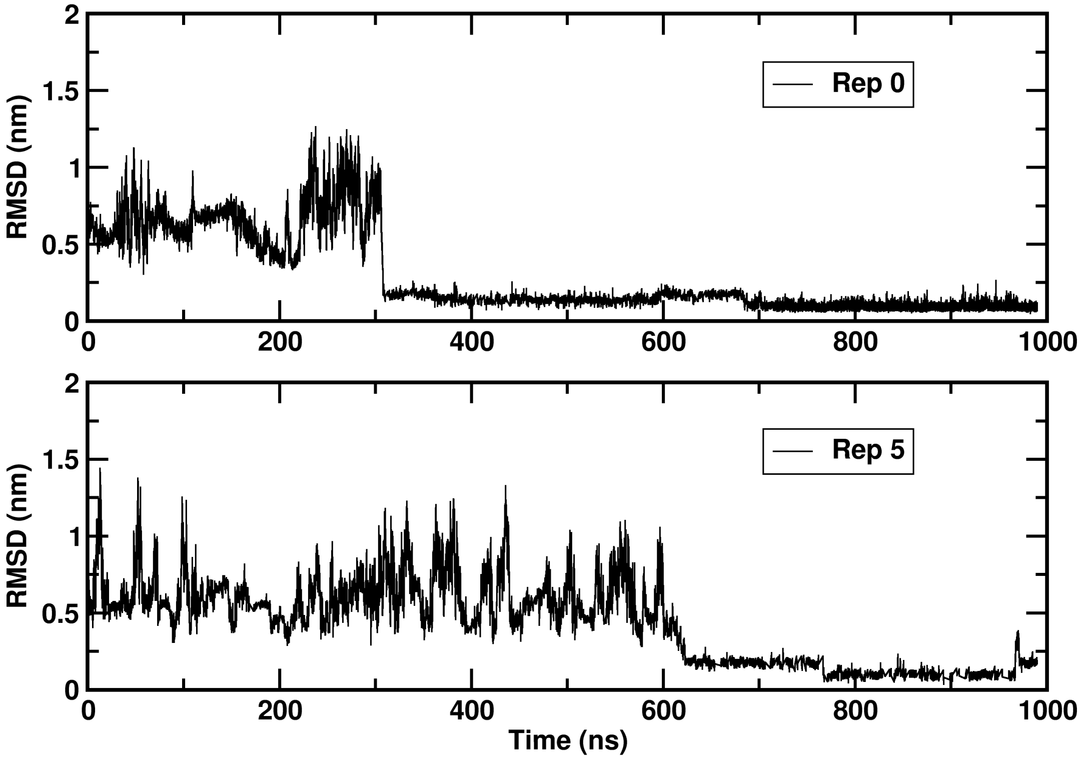

Supplementary Figure 5: Time evolutions of backbone RMSD of REST2-generated TRP-cage structures with reference to the corresponding NMR structure (1l2y), shown for the only two replicas that are successfully folded.

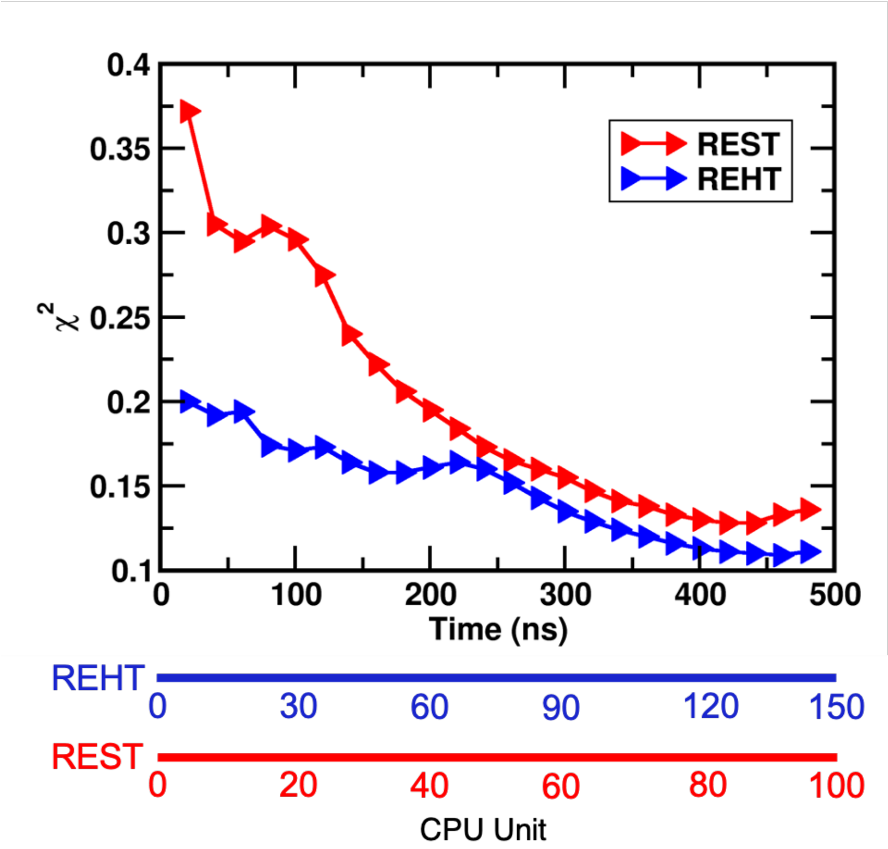

Supplementary Figure 6: Convergence of sampling in physiologically relevant base replica of Trp-cage with REST2 (red) and REHT (blue) methods. The CPU time including the contributions from all replicas is provided in additional X-axes. The figure clearly indicates that the results obtained from REHT converges faster and better than the REST2 simulations.

Supplementary Note 1: A quantitative comparison of ergodicity with REST2 and REHT simulations

We assessed the convergence of REST2 and REHT at the physiologically relevant base replicas (@Temp 300K) similar to that suggested by Dave Thirumalai's group [2] and Bruce Berne's group[3]. Towards this, the ensemble at the base replica is split into two equal parts, one at the start (part A) and other at the end (part B) of the total simulation (leaving a gap of 50ns between them). The two parts of the simulation represents the two independent trajectories started from different conformations of protein. The ergodicity is then measured by comparing the two simulations as follows: The conformational landscape represented by the two CVs is discretized into m*n uniform bins. The population in each of the (i,j)th bin ($P_{i,j}$) is compared between the two parts of the simulations A and B and the overall difference is measured using χ^2 parameter defined as: $\chi^2 = \sqrt{\sum_{i=1;j=1}^{m,n} (P_{i,j}^A - P_{i,j}^B)^2}$. If the sampling method is ergodic, the χ^2 should decay to zero.

Supplementary Figures 6 and 14 depict the χ^2 as the increasing length of simulation time. The figure indicates that the REHT converges faster than the REST2 in both Trp-cage and His-5 simulations. The difference in convergence between the two methods is more accentuated in His-5 simulation.

A more rigorous measure of convergence in replica exchange simulations is to check the convergence of distributions sampled in the independent time continuous replicas. This has been shown earlier with alanine-di-peptide system[4], where the energy landscape is much smoother. This analysis is extremely challenging on very rugged realistic protein landscapes and demanding from sampling point of view. We performed these analyses on the time-continuous replicas of Trp-Cage (Supplementary Figure 7). The distributions of RMSD to the native structure reveal similar trends for replicas that did not explore the folded state, whereas the replicas that fold, sample distributions that are different than each other. This is observed for both REST2 and REHT methods. We anticipate that because of the complex rugged nature of the energy landscape and the existence of multiple pathways to folding. Achieving convergence of protein folding by sampling all the kinetic pathways in a single replica is a challenging task with the current level of simulation time in replica exchange simulations.[5] We would also like to emphasize that in fact the current state-of-the-art REX methods such as REST2 and gREST explored independent folding in one or two replicas leaving most of the replicas predominantly unfolded.[1] In that respect, the REHT definitely does a far better job by exploring the folding independently in six different replicas. We assume extending the simulation longer or increasing the temperature of water further may help achieving this.

Notably, the converged sampling of a flatter landscape in intrinsically disordered His-5 is easily accessible with the REHT method compared to REST2, as will be discussed in Supplementary Figures 14 and 15.

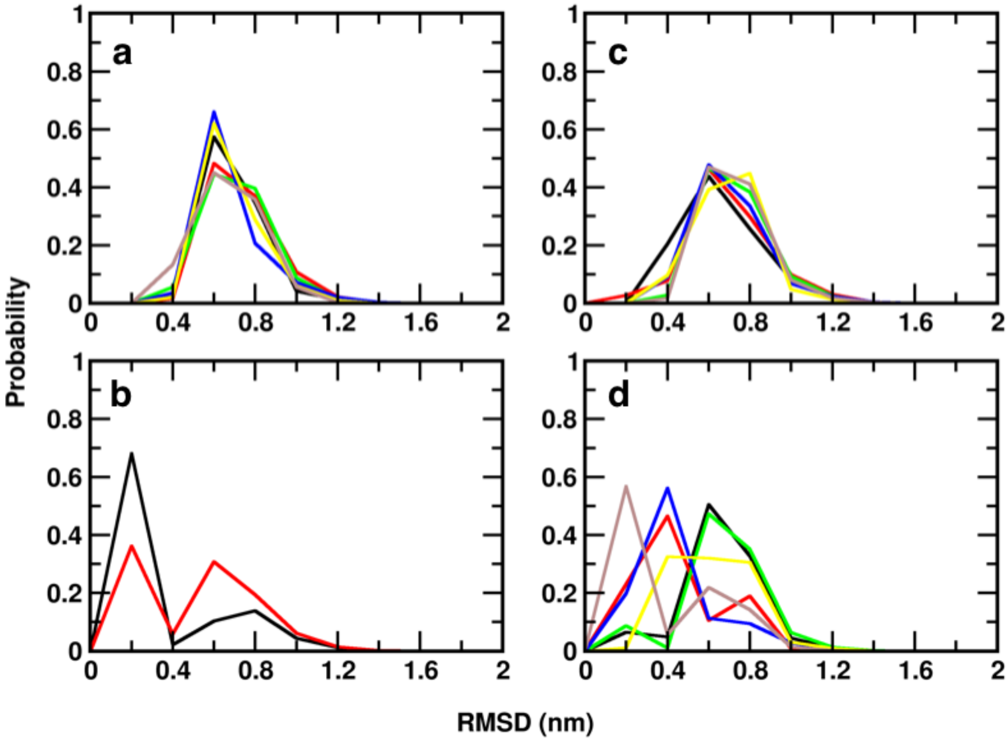

Supplementary Figure 7: Conformational distributions of Trp-cage, plotted with the RMSD to native structure as collective variable in the time continuous replicas of REST2 (**a** and **b**) and REHT (**c** and **d**) ensembles. The distributions for the replicas that have not explored the native folded conformations in REST2 and REHT are shown in **a**) and **c**) respectively. Whereas the distributions for the replicas that explored the folding are shown in **b**) and **d**) for the REST2 and REHT simulations.

Supplementary Note 2: Dihedral switch in Alanine dipeptide

Being the simplest biomolecular model that possesses multiple conformational basins, alanine dipeptide serves as one of the first choices in enhanced sampling literature to study the barrier crossing events. The peptide exhibits three basins across the phi and psi dihedral space. We employed both the conventional REST2 and the novel REHT methods to sample the switching between these dihedral basins of the peptide. The sampled basins across the phi and psi space at different time intervals are illustrated for the lowest temperature replica in Supplementary Figure 4. While REST2 can sample all the basins within 4 ns timescale, REHT required 6 ns to completely sample this dihedral space. However, the relatively slow transitioning phi angle is frequently sampled in the latter method than the former method (Supplementary Figure 5). Moreover, the transition was sampled by 4 out of 5 replicas in REHT method, but only once by a single replica out of three in conventional REST2 method. This result suggests that optimal heating of both solute

and solvent allows effective crossing of the energy barrier and facilitates frequent transition in the conformational space.

a

b

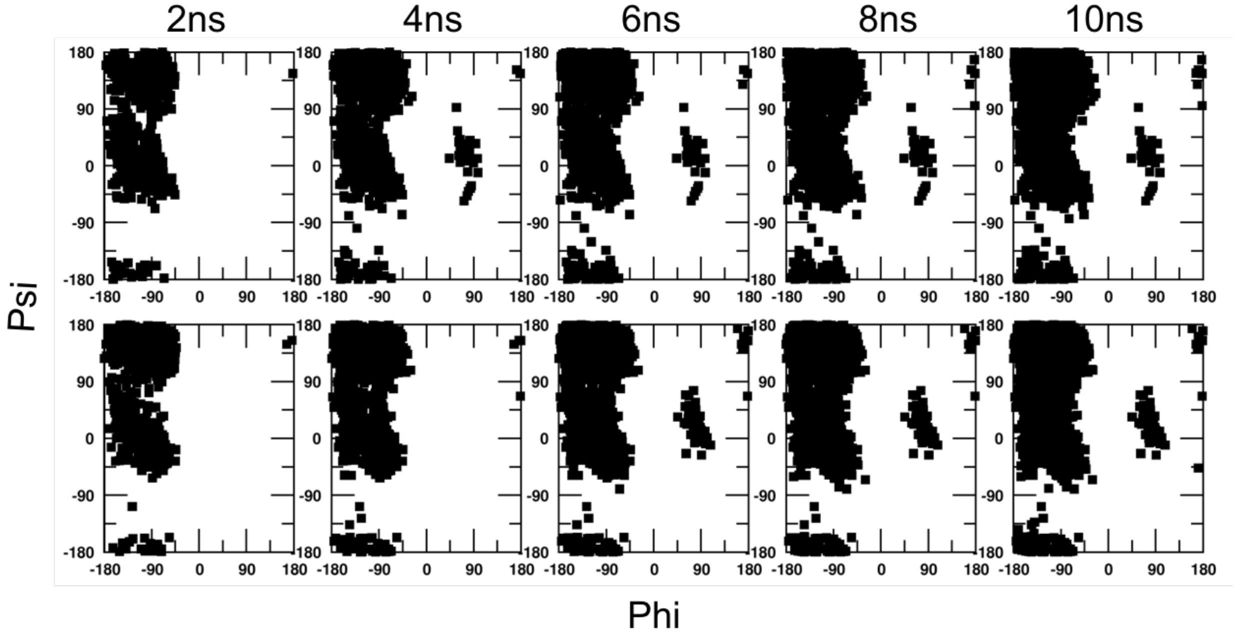

Supplementary Figure 8: Ramachandran map of alanine dipeptide. The average distribution of dihedral angles of Alanine dipeptide obtained at various time points of REST2 (**a**) and REHT (**b**) simulations.

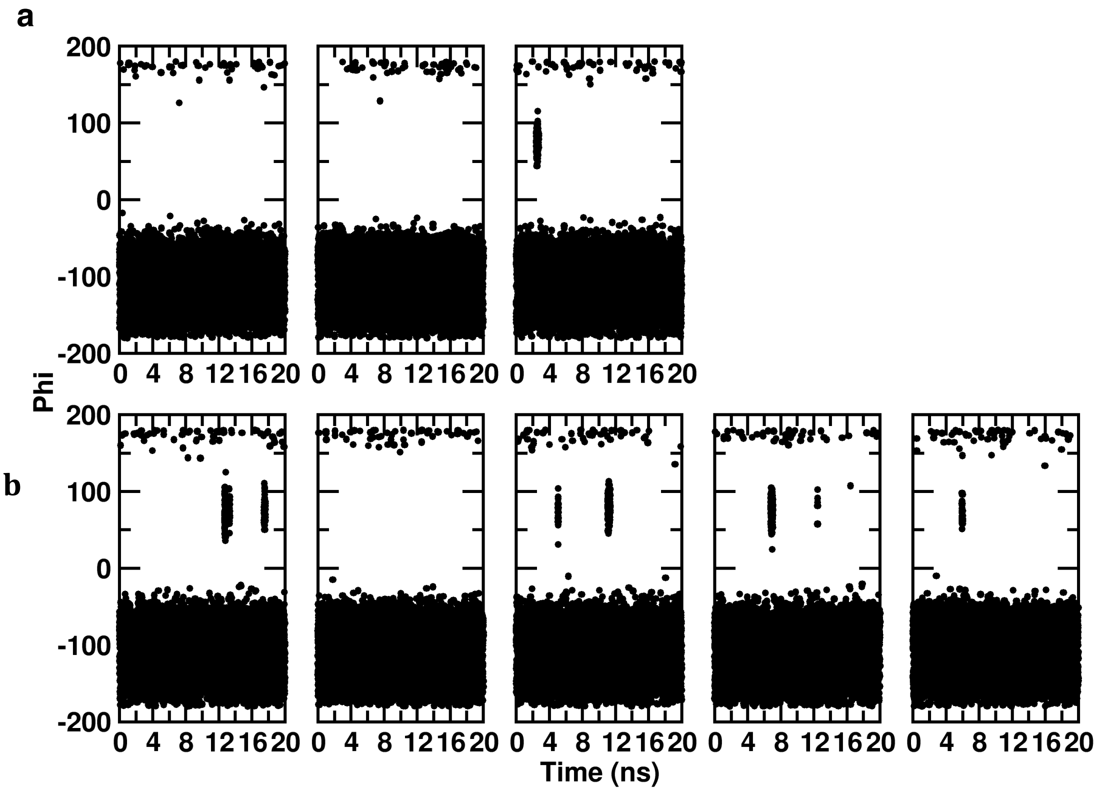

Supplementary Figure 9: Time evolution of slow transitioning Phi angle of alanine dipeptide simulated using **a**) three replicas in REST2 simulations and **b**) five replicas in REHT simulations.

a

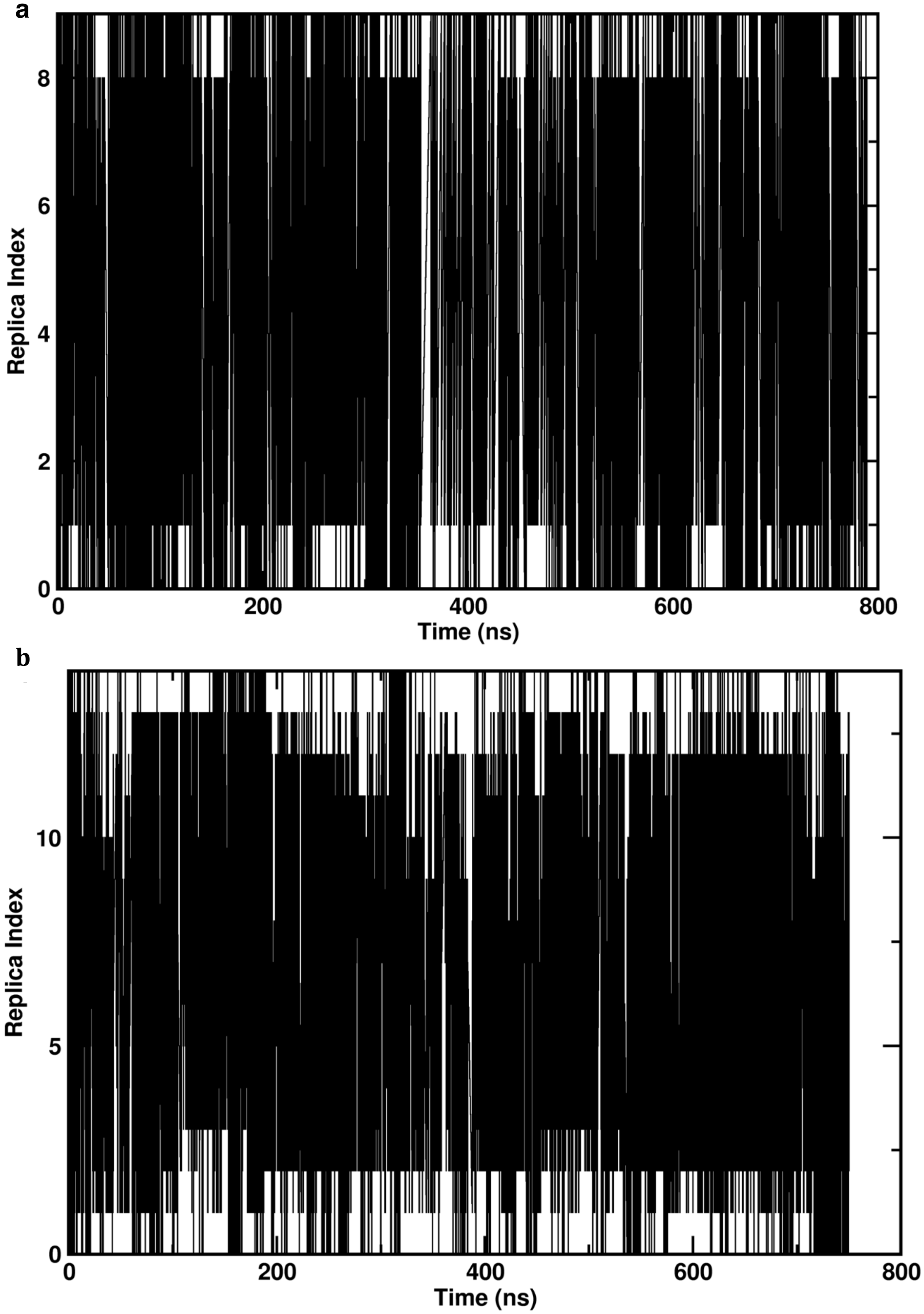

b

Supplementary Figure 10: Exchange of base replica (Rep0) of His-5 system across the entire replica space as a function of time in **a**) REST2 and **b**) REHT simulation.

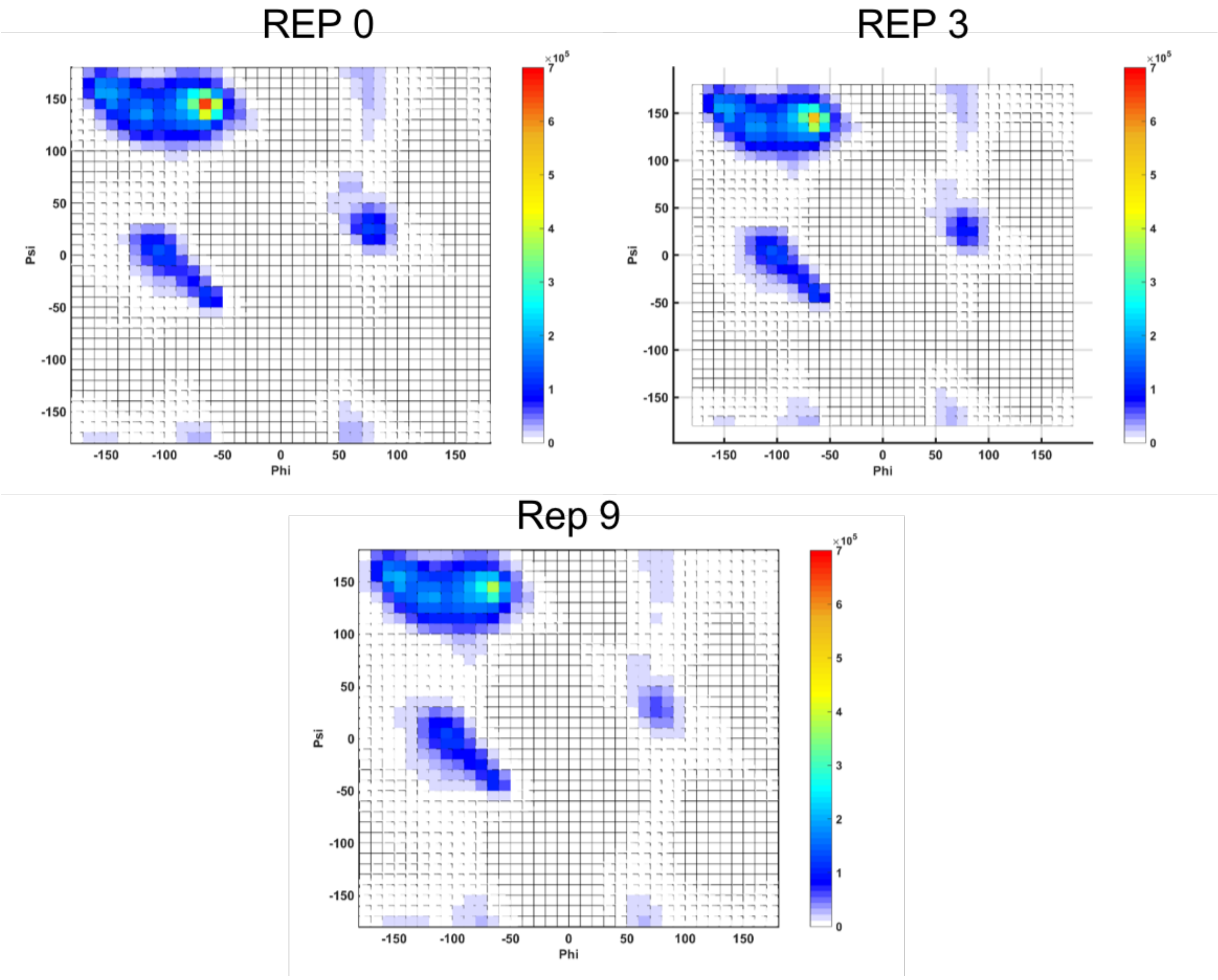

Supplementary Figure 11: Distribution of dihedral angles projected on the Ramachandran map for all residues of Histatin-5 structures obtaned from REHT simulations. The plot is shown for three replicas (replicas 0, 3 and 9) arranged in increasing order of their respective temperatures (300K, 308K, 325K)

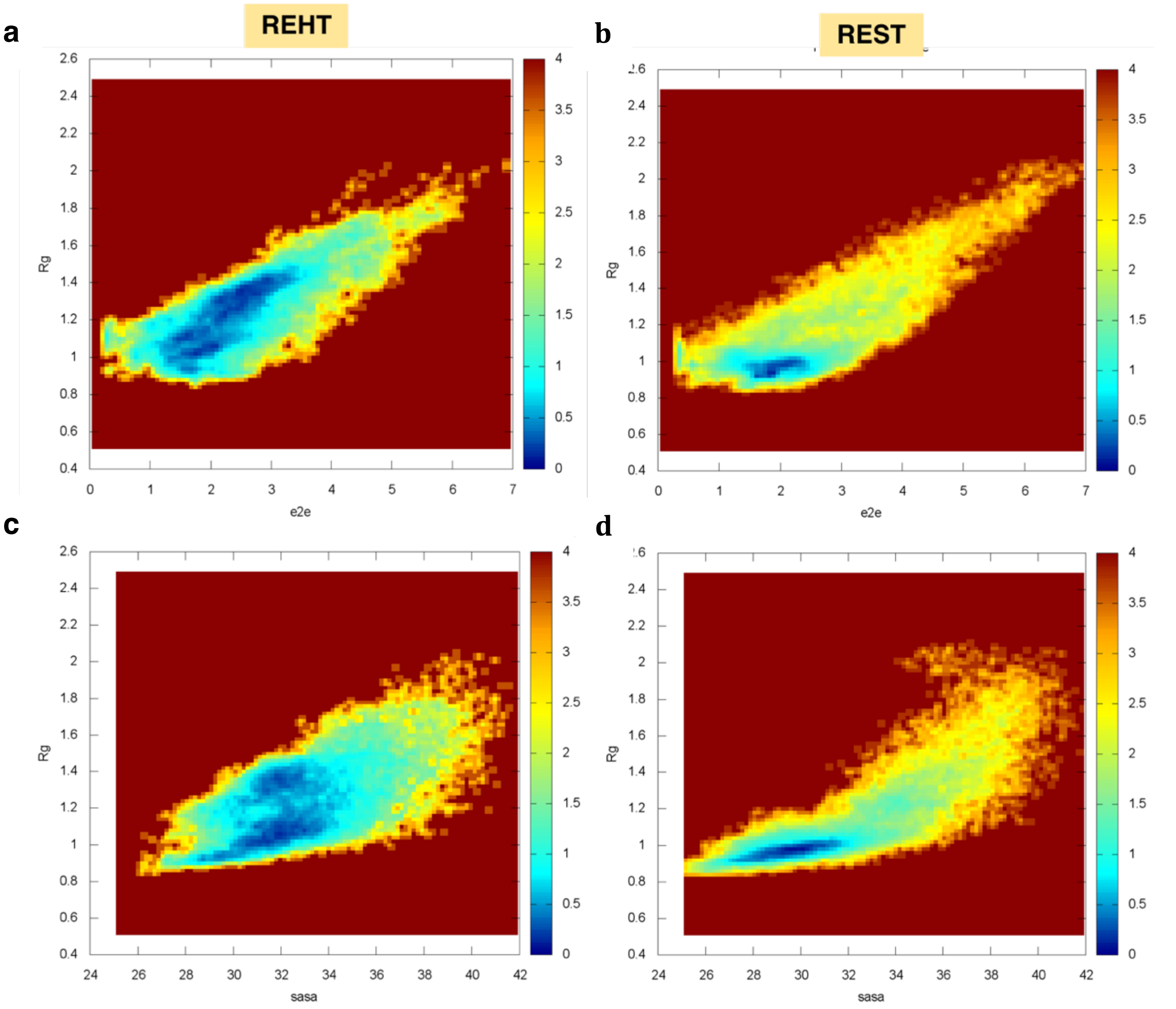

Supplementary Figure 12: 2D representation of free energy landscape of Histatin-5 ensemble collected at the base replica of REHT (**a, c**) and REST (**b, d**) simulations plotted as a function of end to end distance (in nm) vs Rg (in nm) and SASA (in nm^2) vs Rg (in nm).

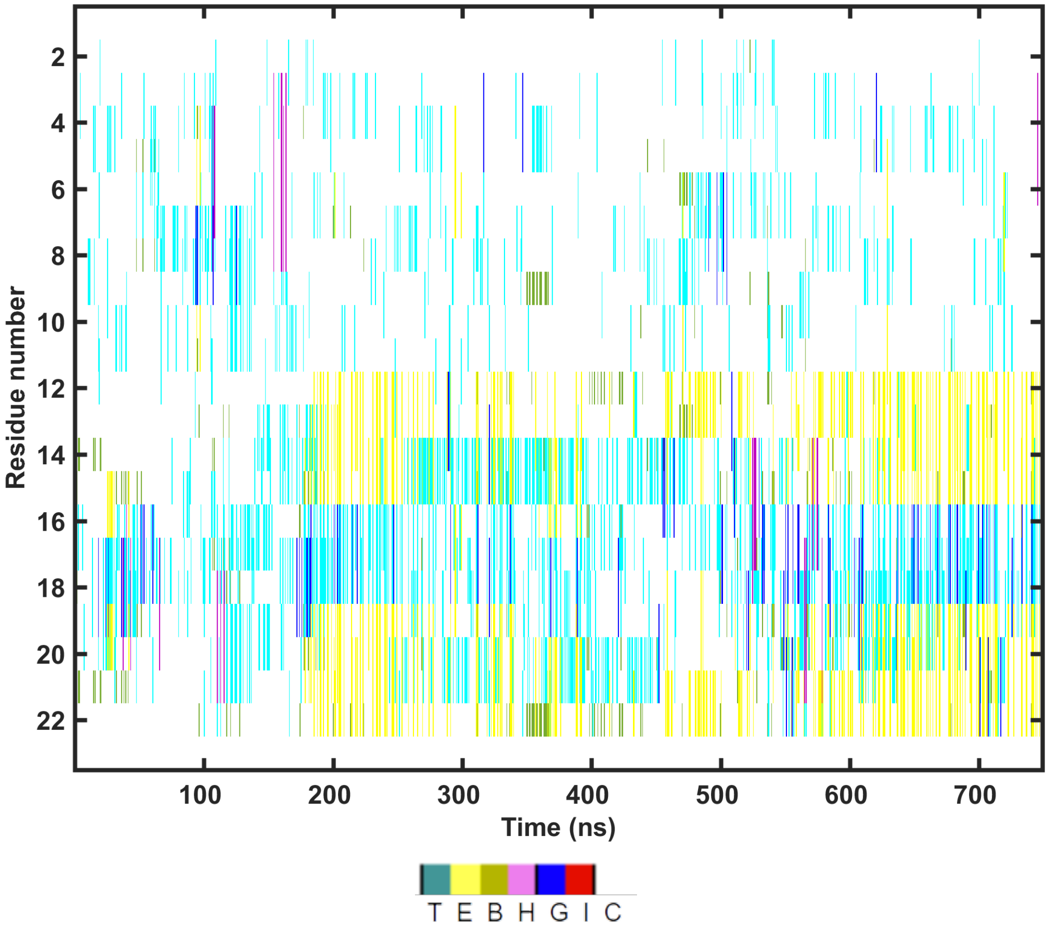

Supplementary Figure 13: Time series of secondary structure of Histatin-5 ensemble collected at the base replica of REHT simulations.

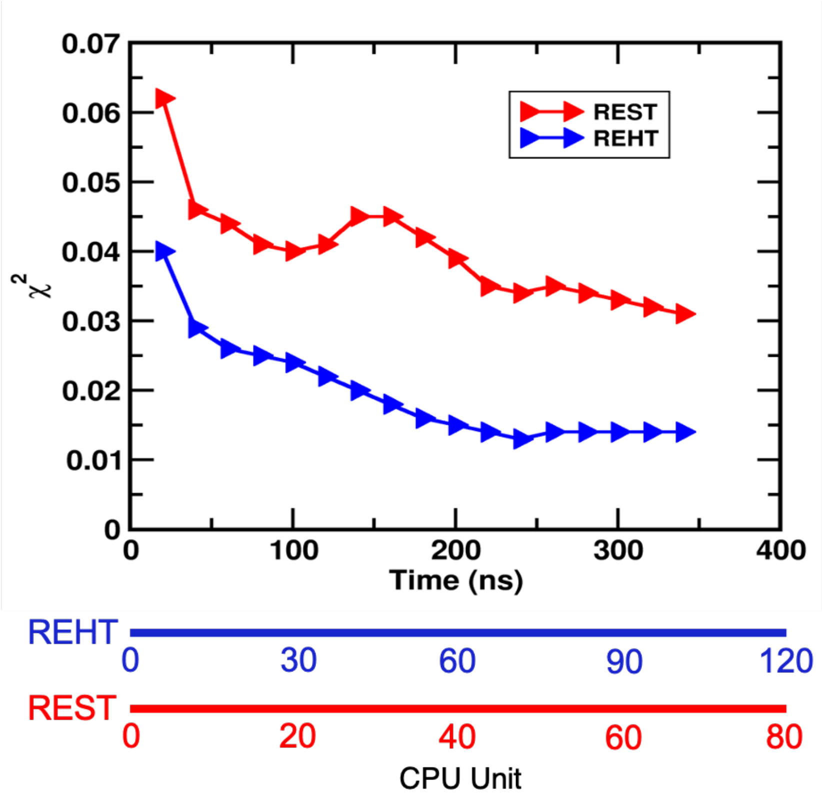

Supplementary Figure 14: Convergence of conformational distributions in His-5 simulated with REST2 (red) and REHT (blue) plotted as a function of simulation length. The CPU time including the contributions from all replicas is provided in additional X-axes.

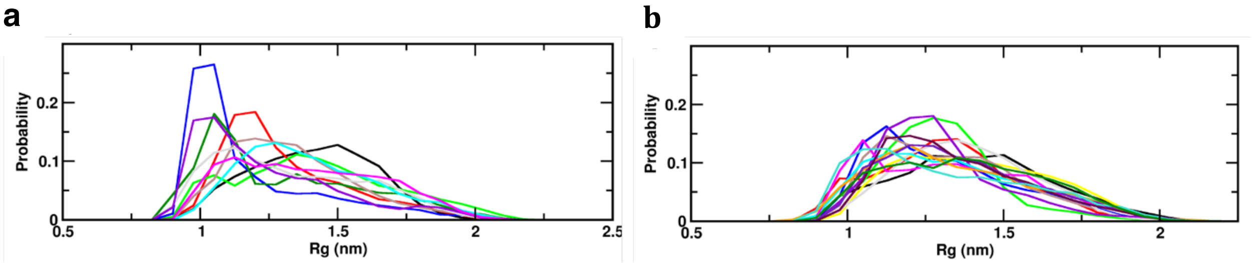

Supplementary Figure 15: Distributions of radius of gyration at different time continuous replicas of **a**) REST2 and **b**) REHT simulations. The plot shows decent overlaps for REHT as compared to REST2. Some of the replicas in REST2 is stuck at compact states (Rg ~ 1nm) resulting in poor exploration of extended states (Rg > 1.3nm). This result provides a compelling evidence that the REST2 is poorly converged and possibly stuck at the entropic barrier resulting in larger variation in the Rg distributions across the replicas. This result is also supported by Supplementary Figure 18.

Supplementary Note 3: Estimation of relative computational cost

Estimation of the relative computational cost between REHT and REST2 should be performed in fully converged simulations. However, the results, as plotted on Supplementary Figures 6 and 14 indicate that the REST2 is not well converged as that of REHT in the timescale simulated for both Trp-cage and His-5 simulations.

We are uncertain how the trend flares out upon extending the REST2 simulation for estimating the relative computational cost. However, extrapolation of the existing data, which assumes that the trends will continue, suggests the REST2 would take about 1000ns/replica in order for attaining the convergence level of 0.1 in TRP-cage (Supplementary Figure 16). On the contrary REHT achieves this in 500ns. In terms of computational cost (CPU units), this is only 1.3 folds reduction in REHT in comparison to REST2 considering all the replicas. Surprisingly, in case of the IDP, His-5, REHT is about 12 folds faster than REST2 (2600ns in REST2 vs 150ns in REHT for achieving a χ^2 value of 0.02 and considering a total of 10 vs 15 replicas used).

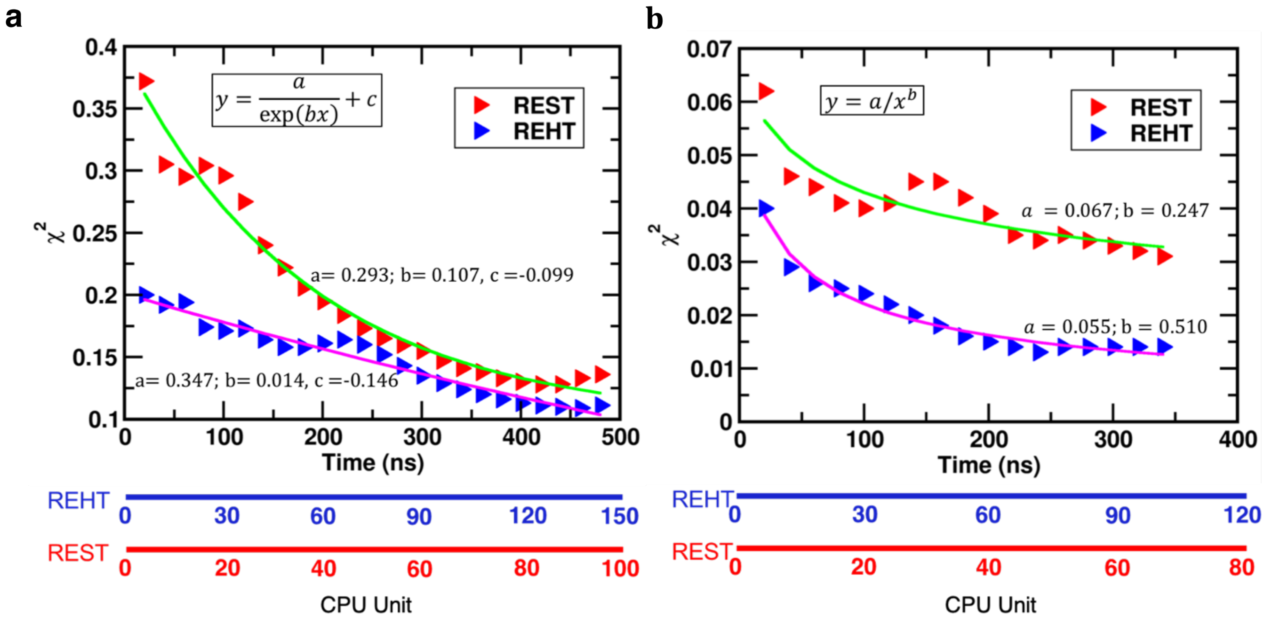

Supplementary Figure 16: Estimating relative computational cost from the convergence decay for **a**) Trp-cage and **b**) His-5 with REST2 (red) and REHT (blue). The best fit lines for each of the curves have been plotted (green and magenta) and their respective functions and parameters are indicated. The CPU time including the contributions from all replicas is provided in additional X-axes.

Supplementary Note 4: Quantification of Heterogeneity of ensemble

To quantify the heterogeneity as suggested by Lyle et.al,[6] each of the conformation in an ensemble is represented as a vector of inter-atomic distances of all C-alpha atoms (V). The distance, D, between two conformational vectors (V_k and V_l) is then computed with the cosine distance defined as $D_{kl} = 1 - V_k . V_l / |V_k||V_l|$. The larger D value indicates more conformational heterogeneity and vice versa. For an ensemble of N conformations, the pairwise distance calculations yield N(N-1)/2 distance values. The distribution of D calculated for His-5 ensemble with REST2 and REHT is plotted in Supplementary Figure 17a. As a control, we also generated the Flory Random Coil (FRC) ensemble for the specified sequence of His-5 using Campari tool and plotted their distance distribution. 5000 conformations were generated with $3*10^7$ Monte Carlo steps after discarding first 500000 steps of equilibration. Our results indicate larger heterogeneity for the REST2 ensemble than for the REHT and FRC, which at face value is quite counterintuitive.

To verify this result, we also used a second heterogeneity measure described by Papoian et.al.[7] According to this metric, the heterogeneity (q) between two conformational vector k and l is computed with the differences in pair distance values ($r_{a,b}$) as follows: $q_{kl} = \frac{1}{N_{pairs}} \sum_{a,b} \exp\left[-\frac{(r_{ab}^k - r_{ab}^l)^2}{2\sigma^2}\right]$, where the σ is a resolution parameter and usually is set to 2Å. Consistent with the cosine distance distribution (Supplementary Figure 17a), the histogram of the pairwise-q (Supplementary Figure 17b) also reveals larger heterogeneity (low q) for REST2 than the REHT and FRC. However, the free energy landscape of His-5 shown with Rg and SASA (Supplementary Figure 12) suggests a confined sampling of compact states in REST2. These conflicting results needed to be reconciled and we anticipated that one of the possible explanations for this inconsistency could have its origin in the "*heterogeneous compact structures*" that the REST2 simulations sample for the His-5 ensemble.

To inspect this, we transformed the cosine distances (D_{kl}) between all pairs of conformations (n*(n-1)/2 values) into a 2-dimensional map of n conformations using multidimensional scaling (MDS). MDS is particularly useful for visualizing the distance matrix in low dimensional space while preserving the between-object distances as much as possible.

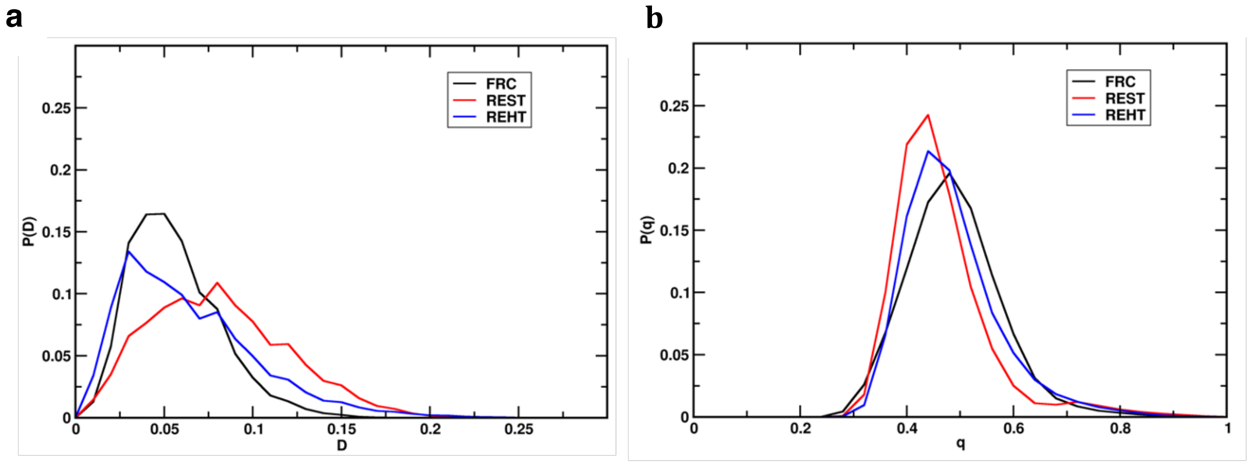

Supplementary Figure 17: Distributions of heterogeneity values calculated using Cosine distance (**a**) and pairwise q-factor (**b**). The heterogeneity of the ensembles generated with the base replicas of REST2 (red), REHT (blue) methods have been compared against the control FRC ensemble (black). The larger D and smaller q represent greater heterogeneity in the ensemble.

a b

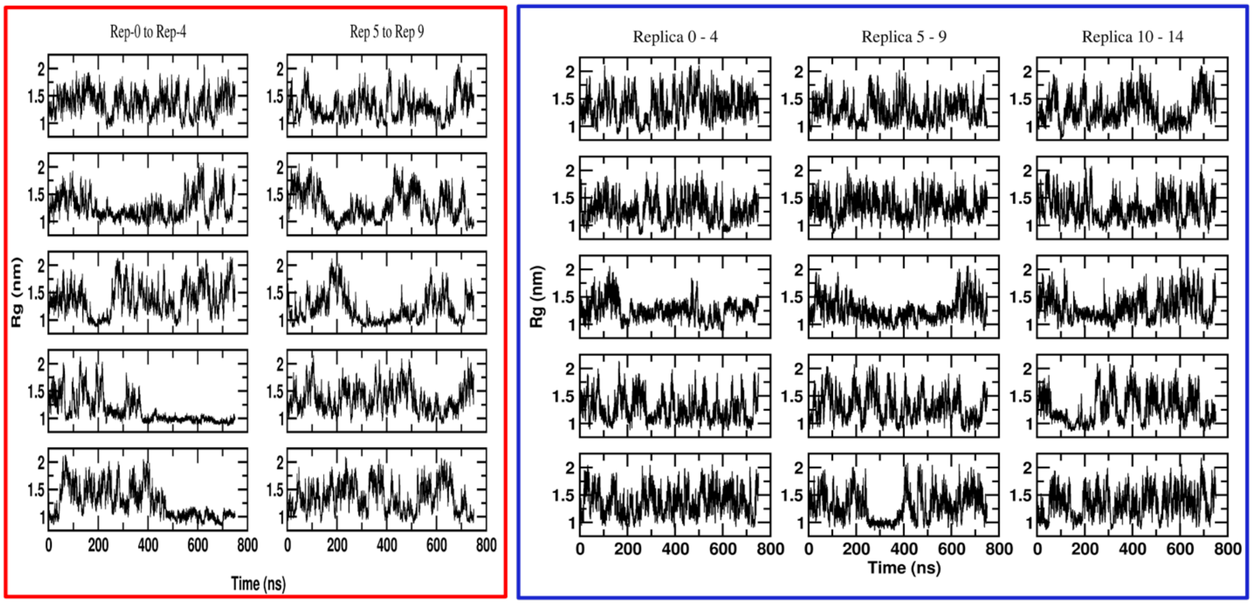

Supplementary Figure 18: Evolution of Rg across the time-continuous replicas of His-5 simulated with REST (**a**) and REHT (**b**) methods. REST shows the trapping of compact states in multiple replicas; whereas, the REHT rapidly explores the dynamically heterogeneous conformations in all the replicas.

a

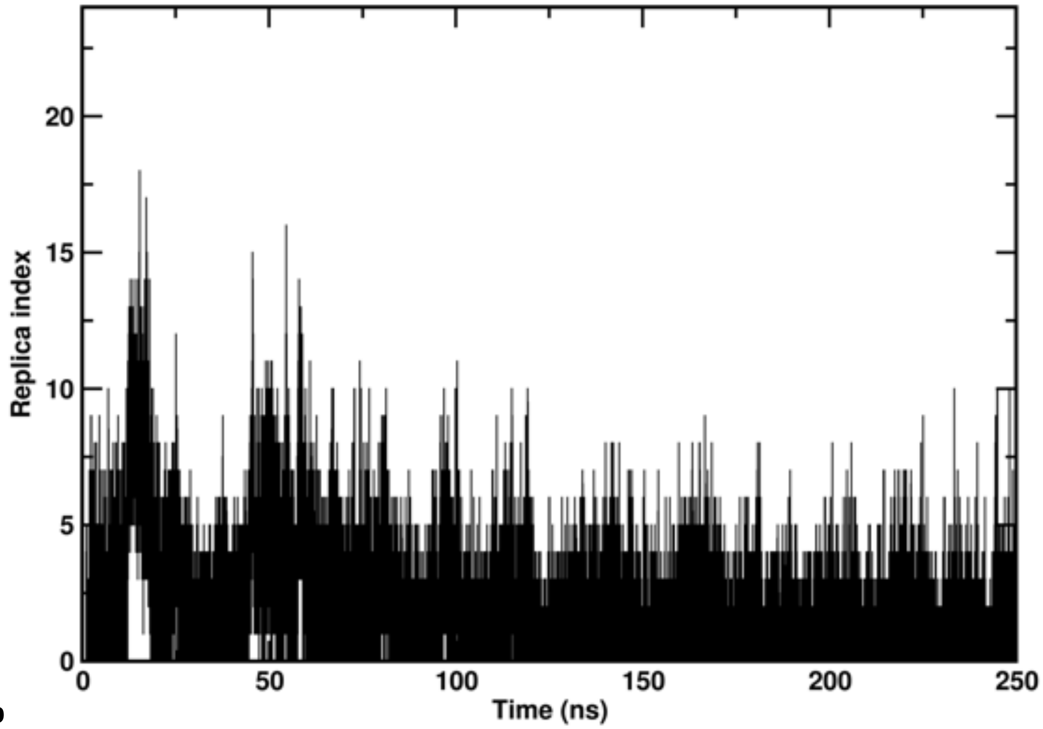

b

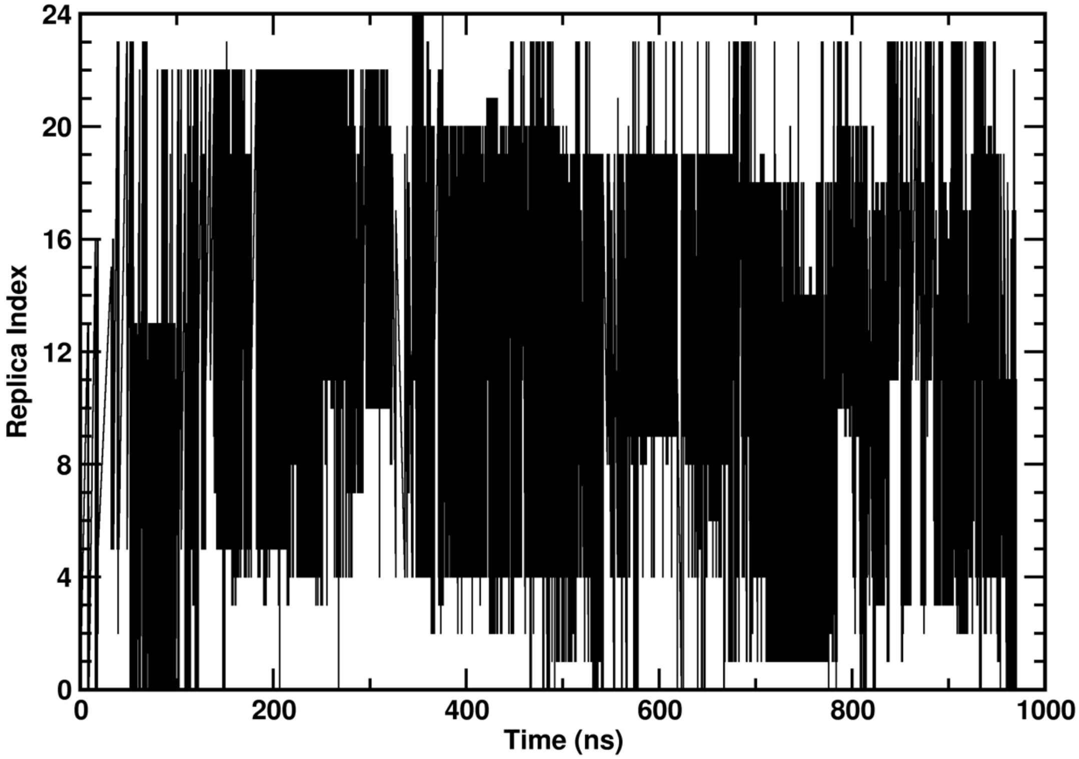

Supplementary Figure 19: Exchange of lowest rank replica across the complete replica state space in RFA-H system simulated with **a**) REST2 and **b**) REHT method.

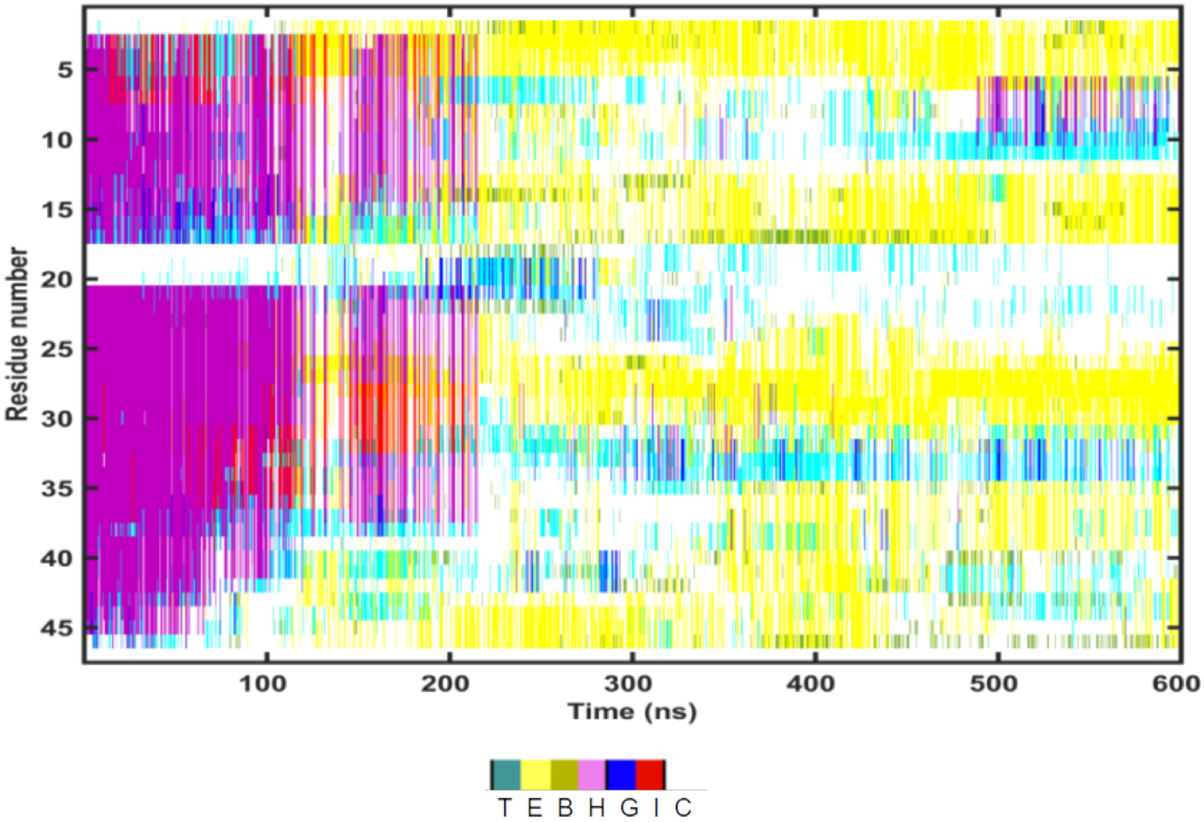

Supplementary Figure 20: Time evolution of secondary structure for the RFA-H ensemble generated from REHT simulations using all- α-helix (2OUG) as starting structure.

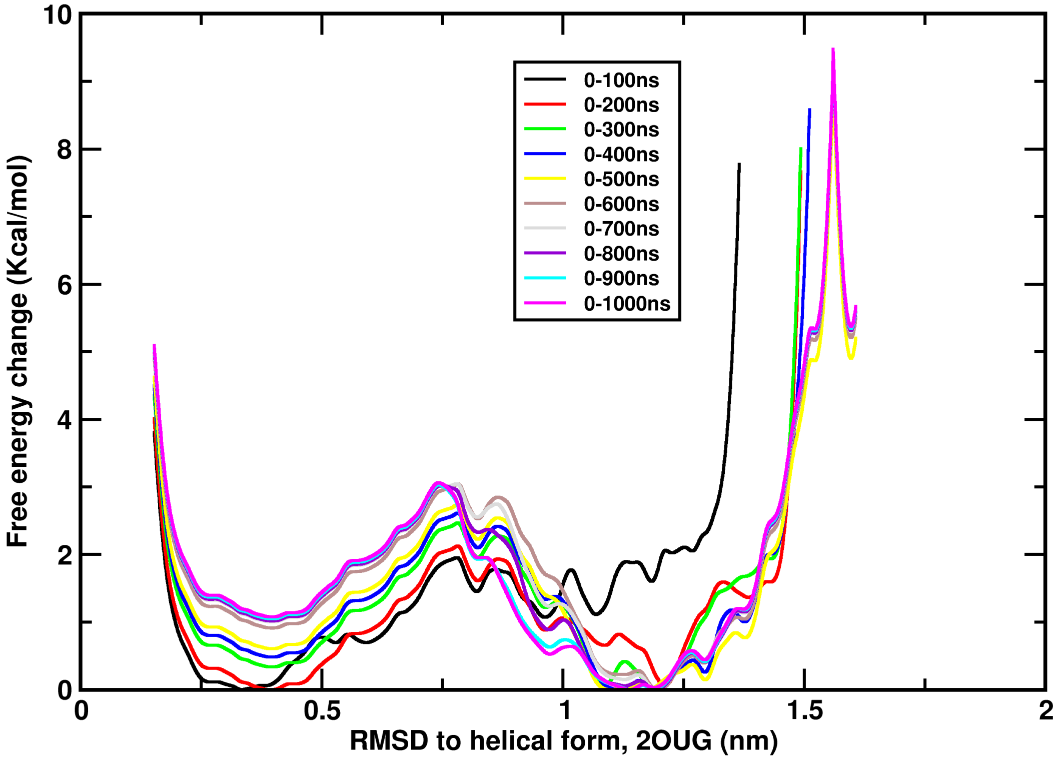

Supplementary Figure 21: Convergence of free energy surface of RFA-H shown along a single reaction coordinate - RMSD with respect to the helical form (pdb ID: 2OUG). The results are shown for different lengths of REHT simulations.

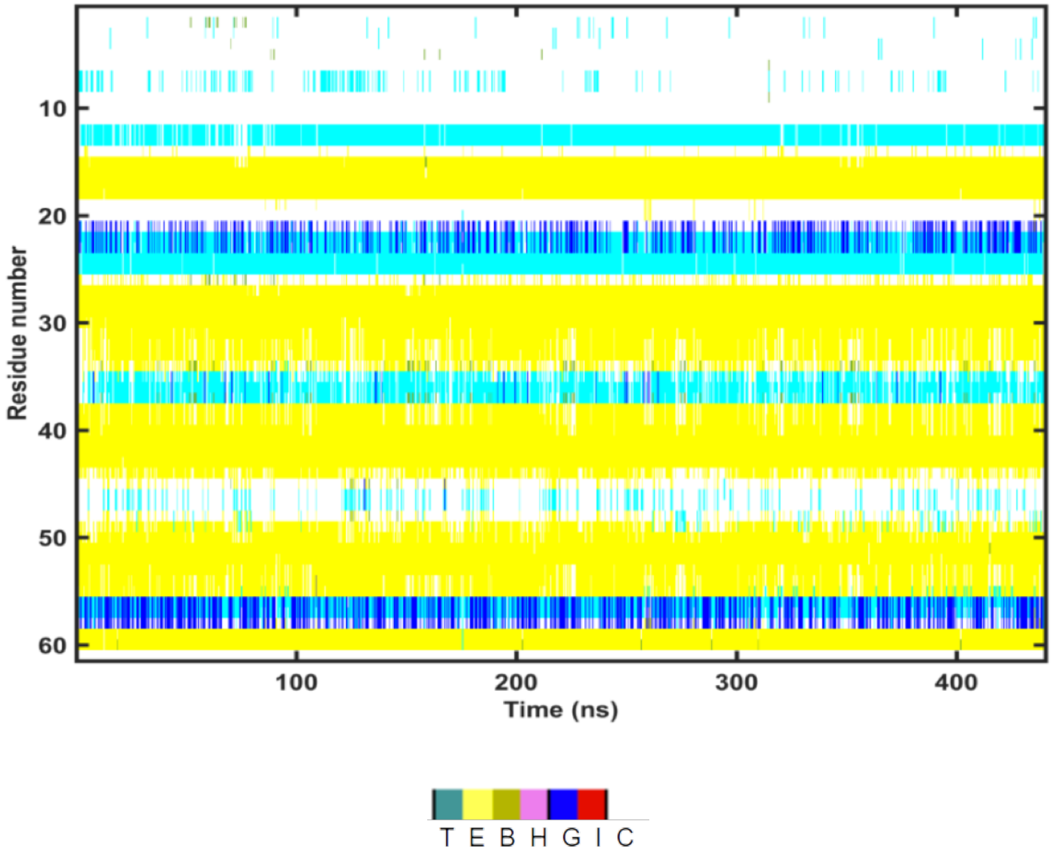

Supplementary Figure 22: Secondary structure of all residues of RFA-H along the time series. The plot corresponds to the lowest rank ensemble (310K replica) of REHT simulation initiated from the experimental beta barrel structure.

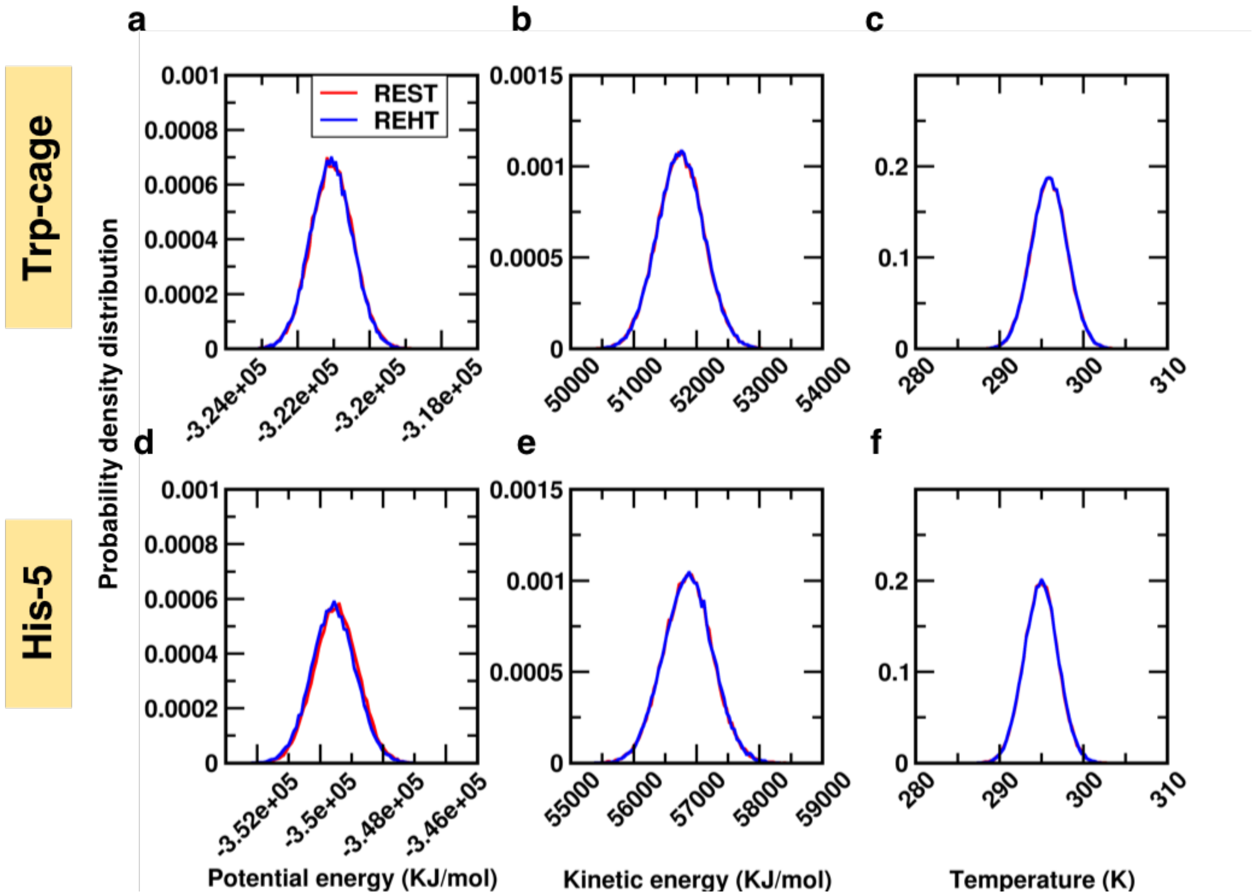

Supplementary Figure 23: Comparison of potential energy (**a, d**), kinetic energy (**b, e**) and Temperature (**c, f**) of water in the base replicas of Trp-cage and His-5 simulations with REST2 (red) and REHT (blue) methods.

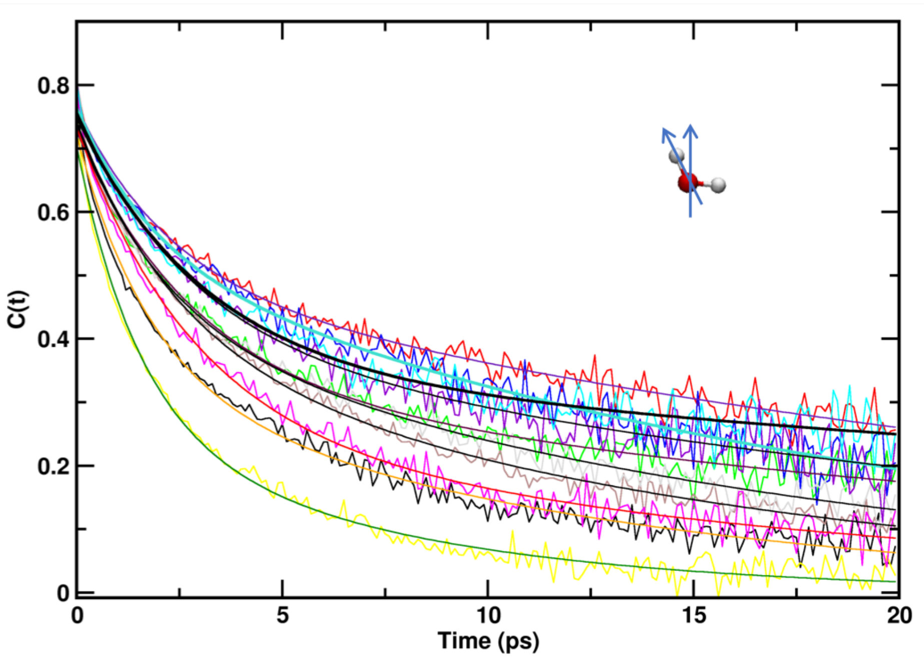

Supplementary Figure 24: Representative curves of water orientation relaxation at various time points of Trp-cage simulation with REHT method. The curves were fitted to biexponential decay ($C_t = \ A\ exp^{-x/\tau_1} + B\ exp^{-x/\tau_2}$) and the fitted curves are shown for each set.

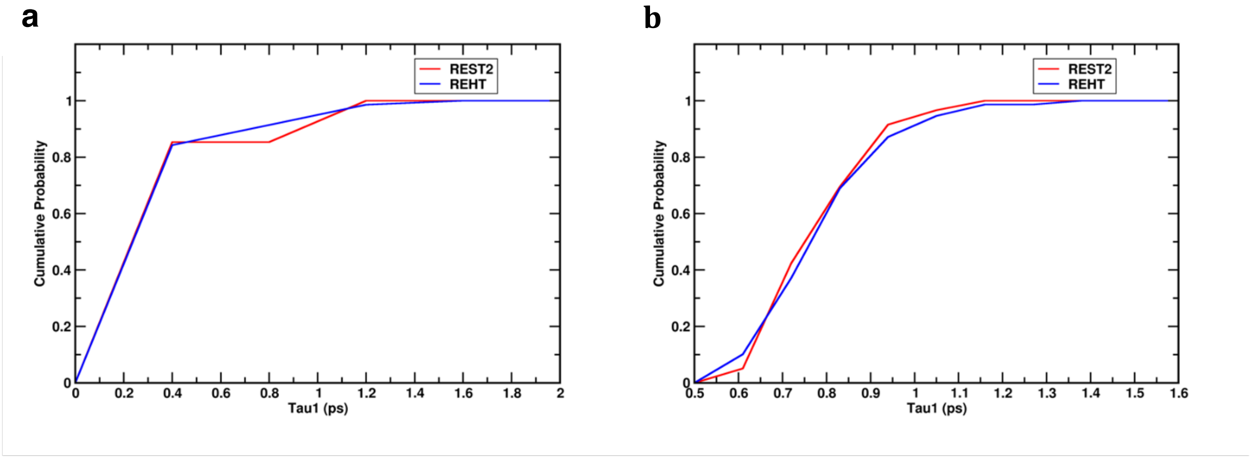

Supplementary Figure 25: Cumulative distribution of fast (τ_1) component of water orientational relaxation decay in REST2 (red) and REHT (blue) simulations of Trp-cage (**a**) and His-5 (**b**).

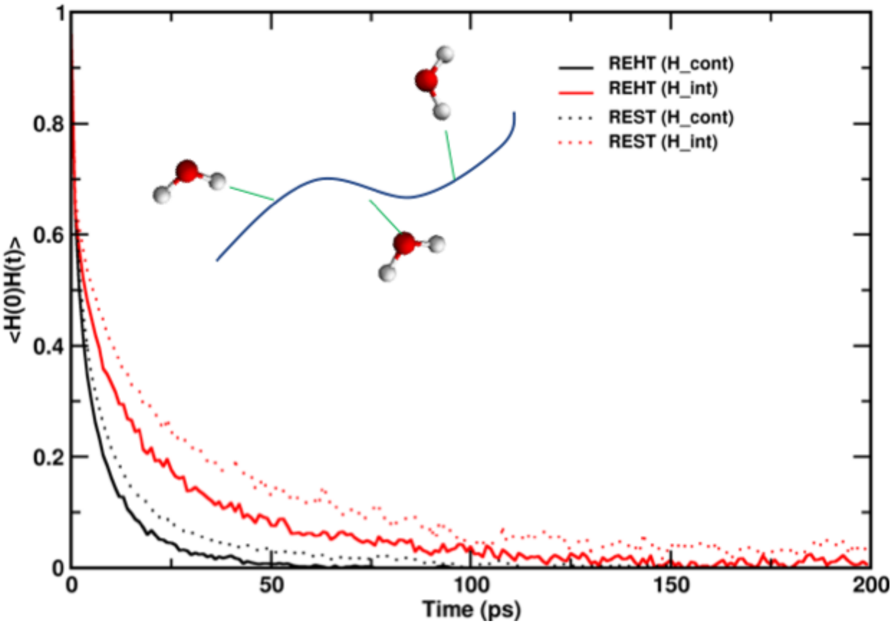

Supplementary Figure 26: Effect of altered hydration dynamics in the lifetime of protein-water H-bonds in Trp-cage folding: Time correlation functions of protein-water H-bonds formed by both continuous (black) and intermittent (red) water molecules are shown. Solid line represents the REHT simulation and dotted line represents the REST2 simulation.

Supplementary Note 5:

Replica exchange simulation methodology

Replica exchange simulations allows for studying equilibrium properties with lesser computational time by its rapid relaxation and improved conformational sampling. Originally developed in Monte-Carlo background (Swendsen and Wang, 1986),[8] the method was later introduced to Molecular dynamics simulations by Sugita and Okomoto in 1999[9] (Temperature replica exchange molecular dynamics (TREM)). The method achieves effective sampling by simulating a series of low and high temperature replicas, while allowing the exchange of configurations at regular intervals. Moreover, due to its stochastic nature of exchanges that ensures the detailed balance, it generates Boltzmann weighted ensemble from which it is straight forward to obtain the thermodynamic averages. The probability of accepting the exchange between replica m and n depends on the difference in the Boltzmann weight factor, that is exponentially related to the difference in energy and temperature (Equation (S1)).

$$P_{acc}^{m\leftrightarrow n}(TREM) = \begin{cases} 1, & \Delta_{nm} \leq 0 \\ \exp(-\Delta_{nm}), & \Delta_{nm} > 0 \end{cases}$$

$$\Delta_{\mathrm{nm}} = (\beta_n - \beta_m)\left[E_q^m - E_q^n\right], \quad \text{(S1)}$$

Where β_n and β_m are reciprocal temperatures ($1/(K_B T_n)$ and $1/(K_B T_m)$ respectively). E_q^m and E_q^n are potential energy of replicas m and n respectively. For a larger system the temperature differences between two replicas should be chosen minimal to yield viable exchange acceptance ratio. This demands large number of replicas to cover a sufficient range of temperatures.

Replica exchange solute tempering (REST2):

Instead of changing the temperature across the replica ladder the advanced replica exchange solute tempering (REST2) method scales the energy function in a particle-wise manner such that the solute is effectively heated up while keeping the water cold. For instance, the potential energy function of replica m is broken down into intramolecular protein interactions (H_{pp}), protein-water interactions (H_{pw}), and water self-interactions (H_{ww}) whose potentials are scaled as shown in Equation (2).

$$H_{REST2}^{m} = \lambda H_{pp} + \sqrt{\lambda}\, H_{pw} + H_{ww} \quad \text{(S2)}$$

Where $\lambda = \frac{\beta_m}{\beta_0}$, in which β_0 and β_m are reciprocal temperatures of 0th and mth replicas. While running all the replicas at the same temperature, the method cancels out the energy difference in water self-interaction energy that otherwise hugely contribute for the poor scaling as in TREM. Thus, the acceptance probability of REST2 as shown in Equation (S3) depends only on the energy differences of intramolecular solute energy and intermolecular energy between protein and water.

$$\Delta_{nm}(REST2) = (\beta_m - \beta_n)\left[H_{pp}(X_n) - H_{pp}(X_m) + \frac{\sqrt{\beta_0}}{\sqrt{\beta_m} + \sqrt{\beta_n}}\left(H_{pw}(X_n) - H_{pw}(X_m)\right)\right] \qquad \text{(S3)}$$

Where, $H_{pp}(X_m)$ and $H_{pp}(X_n)$ indicates the intramolecular energy of protein solute in mth and nth replicas. $H_{pw}(X_m)$ and $H_{pw}(X_n)$ are interaction energies of protein and water in the two replicas. β_m, β_n and β_0 are reciprocal temperatures of replicas m, n and 0th replicas respectively.

Replica exchange hybrid tempering (REHT):

Though the REST2 claims to be efficient for studying larger proteins including that of IDPs as shown recently by Shrestha et.al, it suffers from inability to explore the complex energy landscape with larger energy barriers. We speculated that this could be due to the imbalance between hot solute and cold solvent that causes differential dynamics of central protein and surrounding bulk water. In general, the biomolecular folding and conformational transition is tightly coupled to its surrounding water dynamics. Hence in this work, we introduce a hybrid method that optimally treats the protein as well as the surrounding water. We achieve this by associating the replicas to different bath temperatures in addition to scaling down the potential function of protein in contrary to REST2. Treating the replicas in such a combination doesn't violate the detailed balance condition. More importantly the method yields expedited protein conformational sampling by allowing efficient rewiring of water. For the viable exchanges the temperature gaps between the adjacent replicas are kept minimal. At the same time the protein is allowed to effectively heated-up to a larger extent by additional REST2 scaling factor.

References:

1. Kamiya, M. & Sugita, Y. Flexible selection of the solute region in replica exchange with solute tempering: Application to protein-folding simulations. *J. Chem. Phys.* **149**, (2018).
2. Thirumalai, D., Mountain, R. D. & Kirkpatrick, T. R. Ergodic behavior in supercooled liquids and in glasses. *Phys. Rev. A, Gen. Phys.* **39**, 3563–3574 (1989).
3. Liu, P., Kim, B., Friesner, R. a & Berne, B. J. Replica exchange with solute tempering: a method for sampling biological systems in explicit water. *Proc. Natl. Acad. Sci. U. S. A.* **102**, 13749–13754 (2005).
4. Gil-Ley, A. & Bussi, G. Enhanced conformational sampling using replica exchange with collective-variable tempering. *J. Chem. Theory Comput.* **11**, 1077–1085 (2015).
5. Paschek, D., Nymeyer, H. & Garcı, A. E. Replica exchange simulation of reversible folding / unfolding of the Trp-cage miniprotein in explicit solvent : On the structure and possible role of internal water. **157**, 524–533 (2007).
6. Lyle, N., Das, R. K. & Pappu, R. V. A quantitative measure for protein conformational heterogeneity. *J. Chem. Phys.* **139**, 121907 (2013).
7. Potoyan, D. A. & Papoian, G. A. Regulation of the H4 tail binding and folding landscapes via Lys-16 acetylation. *Proc. Natl. Acad. Sci. U. S. A.* **109**, 17857–17862 (2012).
8. Tyler, R. C., Murray, N. J., Peterson, F. C. & Volkman, B. F. Native-State Interconversion of a Metamorphic Protein Requires Global Unfolding. *Biochemistry* **50**, 7077–7079 (2011).

9. Sugita, Y. & Okamoto, Y. Replica-exchange molecular dynamics method for protein folding. *Chem. phys Lett.* **314**, 296–297 (1999).
